# Higher serum ascorbic acid levels are associated with lower depression prevalence in US adults: a case-control study

**DOI:** 10.3389/fnut.2024.1324835

**Published:** 2024-01-26

**Authors:** Mengyuan Chen, Haolong Luo, Yan Han, Yuanhong Li, Li Zhou, Xiangmei Ren

**Affiliations:** ^1^Department of Nutrition, School of Public Health, Xuzhou Medical University, Xuzhou, China; ^2^Key Laboratory of Human Genetics and Environmental Medicine, Key Lab of Environment and Health, Xuzhou Medical University, Xuzhou, China

**Keywords:** serum ascorbic acid, depression, propensity score matching, restricted cubic spline, NHANES

## Abstract

**Background:**

Recent studies have shown that a higher intake of ascorbic acid was associated with a lower prevalence of depression. Nevertheless, the recall bias was common in dietary surveys in these studies, and it was ignored that there were differences in the absorption and utilization of ascorbic acid in the body. Hence, we aim to investigate the association between serum ascorbic acid levels and the prevalence of depression in US adults.

**Methods:**

A total of 3,404 participants from the 2017–2018 National Health and Nutrition Examination Survey (NHANES) that underwent measurement of the Patient Health Questionnaire-9 (PHQ-9) scores and serum levels of ascorbic acid. Propensity Score Matching (PSM) successfully established a case–control study, comprising 299 participants diagnosed with depression and 1,107 as controls. We used binary logistic regression to estimate odds ratios (ORs) and 95% confidence intervals (CIs) to explore associated risk factors for depression. Restricted cubic splines (RCS) were used to show the nonlinear relationship between serum ascorbic acid levels and the prevalence of depression.

**Results:**

The prevalence of depression was approximately 8.8%, with a median serum ascorbic acid level of 49.9 (36.0, 67.0) μmol/L. Results revealed that the serum ascorbic acid levels of depressed patients were significantly lower than those of non-depressed individuals (42.97 VS 52.97 μmol/L). The baseline data indicated that as serum ascorbic acid levels increased from Quartile 1 (Q_1_) to Quartile 4 (Q_4_), the depression prevalence decreased from 12.0 to 5.4% (*p* < 0.05). The results of the chi-square test after PSM showed that serum ascorbic acid was still statistically significant (*p* < 0.001) with the prevalence of depression. Forest plot showed that compared with the Q_1_ of serum ascorbic acid level, the OR and 95%CI of depression prevalence in Q_4_ was 0.42 (0.30 ~ 0.61), and the adjusted OR and 95%CI of depressive prevalence was 0.49 (0.33 ~ 0.73). RCS models showed an L-shaped nonlinear relationship (P for nonlinearity <0.05) between serum ascorbic acid and depression.

**Conclusion:**

Our results suggested that higher serum ascorbic acid levels are associated with a reduced prevalence of depression.

## 1 Introduction

Depression is one of the top 25 leading causes of the global burden (1). The COVID-19 pandemic had further compounded the problem. During the period from April to September 2020, the prevalence of positive screens for anxiety and depression among US adults surged to three to four times the rate observed in 2019 (2). It amplified the already significant healthcare burden linked to treatment-resistant depression, which constituted $25.8 billion (56.6%) and added $8.7 billion (47.7%) to the unemployment-related load (3). The etiology and pathophysiology of depression are intricate, encompassing multiple factors, such as psychological, social, biochemical, and genetic aspects (4). Recently, the role of diet has been gaining attention among the modifiable factors associated with depression. Studies had observed that a higher total intake of antioxidants was significantly associated with lower odds of depression (5, 6).

Ascorbic acid (AA) is a vital water-soluble vitamin with potent antioxidant properties, capable of capturing and neutralizing free radicals, thus safeguarding cells against oxidative stress-induced damage (7). The findings of a study indicate that individuals in the highest quartile of dietary antioxidant intake, including vitamin C, exhibit a reduced risk for depression compared to those in the lowest quartile. It is worth noting that augmenting the consumption of dietary antioxidants may serve as a preventive measure against depression (8). Serum ascorbic acid refers to the presence of AA compounds in the human blood plasma, reflecting the body’s level of AA reserves and providing information about the specific status of AA in the body at a particular time. However, a limitation of current studies on ascorbic acid and depression lies in the utilization of dietary review to assess the intake of AA, which may introduce recall bias and overlook variations in its absorption and utilization within the human body (6, 9, 10).

From a nutritional perspective, the serum ascorbic acid content is not always directly related to dietary intake (10, 11). As a valuable biological marker, serum ascorbic acid levels provide comprehensive information that aids in understanding an individual’s specific ascorbic acid status. Hence, numerous studies had extensively explored the association between serum ascorbic acid levels and various diseases, encompassing conditions such as cardiovascular diseases, gallbladder diseases, and kidney stones (12–14). Such studies highlighted the importance of understanding serum ascorbic acid levels in assessing the metabolism and utilization of AA in the human body.

Therefore, the objective of this study is to investigate the correlation between serum levels of ascorbic acid and the prevalence of depression from a serum status perspective, aiming to offer novel insights for depression prevention and treatment.

## 2 Methods

### 2.1 Data source

The National Health and Nutrition Examination Survey (NHANES), conducted by the National Center for Health Statistics, employs a complex, stratified, multistage probability cluster design to comprehensively assess the health and nutritional status of the non-institutionalized civilian population in the United States. This cross-sectional survey encompasses demographic, dietary, physical examination, and questionnaire data. Details on the NHANES methodology and analysis guidelines are readily available. The study protocol was also approved by the Institutional Review Board of the Centers for Disease Control, and all participants provided written informed consent.

**Figure 1:**
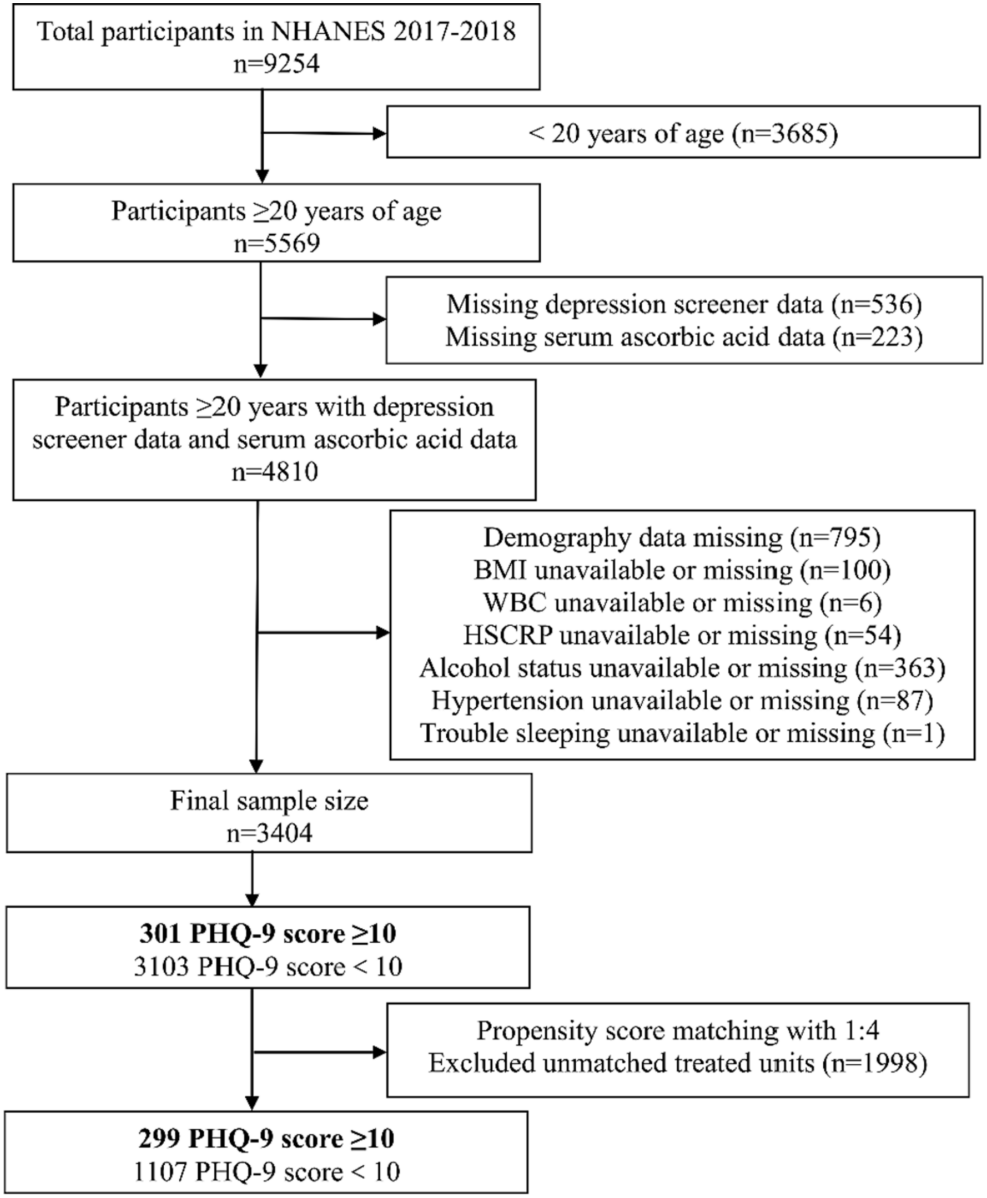
Participant screening flowchart based on the NHANES database for the serum ascorbic acid and depression relationship in 2017–2018.

For this study, a total of 9,254 participants from the 2017–2018 NHANES were originally selected as the study population. Participants under the age of 20 years (*n* = 3,685) were excluded. Subsequently, those with incomplete data on PHQ-9 scores (*n* = 536) and serum ascorbic acid levels (*n* = 233) were also excluded. Other exclusion criteria were as follows: (1) Lack of demographic data (*n* = 795); (2) Lack of Body Mass Index (BMI) data (*n* = 100); (3) Lack of White Blood Cell (WBC) count data (*n* = 6); (4) Lack of High-Sensitivity C-Reactive Protein (HSCRP) data (*n* = 54); (5) Lack of alcohol consumption data (*n* = 363); (6) Lack of hypertension status data (*n* = 87); (7) Lack of sleep problem status data (*n* = 1). After the screening process, a total of 3,404 participants were ultimately enrolled in the study. A flowchart depicting the screening process is presented in Figure 1.

### 2.2 Measurement and definition of variables

This study adopted a semi-structured interview format and utilized the PHQ-9 to assess the presence of depression by evaluating the frequency of depressive symptoms over the past two weeks. The screening tool adhered to the diagnostic criteria for DSM-IV depression and assessed depressive symptoms using a four-point scale for each symptom item: “not at all,” “several days,” “more than half the days,” and “nearly every day,” with corresponding scores ranging from 0 to 3. This resulted in a total score ranging from 0–27 (15, 16). Based on a meta-analysis covering 29 studies with a total of 6,725 participants, the sensitivity of this screening tool in studies utilizing semi-structured interviews was 0.88 (95% CI: 0.83 ~ 0.92), and the specificity was 0.85 (95% CI: 0.82 ~ 0.88). A critical score of 10 or higher maximized the combined sensitivity and specificity (17). Therefore, in this study, participants with a PHQ-9 total score of ≥10 were classified as having clinically relevant depressive symptoms. Serum ascorbic acid levels were measured using isocratic high-performance liquid chromatography at the Centers for Disease Control and Prevention in Atlanta, GA. Detailed instructions for sample collection and processing was available in the NHANES Laboratory Procedure Manual (18).

Participants were classified as having hypertension if they met either of the following criteria in the physical examination data: average systolic blood pressure (SBP) ≥140 mmHg or average diastolic blood pressure (DBP) ≥90 mmHg (19). Participants were confirmed to have diabetes if they answered “yes” to the question, “Has a doctor or health professional ever told you that you have diabetes or high blood sugar?” (20). The determination of trouble sleeping followed a similar course. Additionally, we categorized smoking status into two groups: never smoked (lifetime smoking of fewer than 100 cigarettes) and current/former smoker (lifetime smoking of 100 or more cigarettes) (21). According to alcohol consumption frequency, alcohol status were classified into four categories: “never,” “no more than once a month,” “no more than once a week,” and “more than once a week” (22).

Furthermore, our research also incorporated the subsequent covariates of interest: gender (Male or Female), age groups (20–39 years, 40–59 years, ≥60 years), race (Mexican-American, other Hispanic, Non-Hispanic white, Non-Hispanic black, and Other race), education levels (Less than 12th grade, High school graduate, Some college or Associate of Arts degree, and College graduate or above), marital status (Married/living with partner, Widowed/divorced/separated, and Never married), household poverty income ratio (PIR, categorized based on quartile intervals with quartile values of 1.24, 2.25, and 3.99) (23), BMI, WBC, and HSCRP (categorized into three intervals: <1, 1–3, and > 3 mg/L) (24).

### 2.3 Statistical analysis

Data extraction and merging for the 2017–2018 NHANES were done with SPSS software (version 23.0). Subsequent data analysis and chart design utilized SPSS software (version 23.0) and R Studio (version 4.1.1). Continuous baseline variables were assessed using Student’s t-test, while categorical variables underwent chi-square analysis. Binary logistic regression was employed to investigate potential depression-related risk factors.

Propensity Score Matching (PSM) is a common technique in observational studies for mitigating selection bias. This method is based on causal inference and involves estimating the propensity scores, which represent the probability of individuals being exposed to the treatment group. By employing this method, PSM facilitated matching between the treatment and control groups. It helped eliminate or reduce the impact of confounding factors, enhancing the internal validity and causal inference of the study, especially in non-randomized experimental conditions (25, 26). The method with a 1:4 nearest neighbor matching algorithm was employed in our research to match participants with and without depression. Thoughtfully chosen covariates including gender, age, race, education levels, marital status, and PIR were used in the matching procedure, which was conducted using R Studio software for the PSM analysis.

Restricted Cubic Spline (RCS) is a statistical technique utilized to model the intricate non-linear connection between continuous exposures and outcomes. It applies a non-linear curve by setting fixed knots and using cubic polynomials for interpolation, resulting in a smooth curve fit. The use of restricted conditions ensured the smoothness of the curve at the boundaries and helped prevent overfitting. RCS was widely applied in epidemiology and biostatistics to study non-linear relationships and dose–response associations (27). In our study, a three-knot RCS model was utilized to further investigate the relationship between serum ascorbic acid levels and the prevalence of depression, both before and after accounting for confounding variables. RCS functions were applied using R Studio software, with a significance threshold of *p* < 0.05 indicating statistical significance.

## 3 Results

### 3.1 Participant characteristics

The study eventually enrolled 3,404 participants, from which a population baseline table was generated based on serum ascorbic acid quartile ranges. Among them, approximately 51.1% were males. The participants’ ages ranged from 20 to 80 years, and they were categorized into three age groups, with individuals aged 60 and above accounting for 37.7% of the total sample. In terms of race, 39.1% identified as non-Hispanic white. Regarding education levels, 34.3% of participants had attained some college education or an associate’s degree. Additionally, 59.4% of participants were either married or living with a partner. The PIR ranged from 0 to 5, and it was divided into four intervals based on quartile ranges, with cutoff values at 1.24, 2.25, and 3.99.

Furthermore, 36.0% of the participants had HSCRP concentrations ranging from 1 to 3 mg/L. The prevalence of depression was 8.8% among the participants. Smokers accounted for 46.5%, while 35.6% reported alcohol consumption less than once a month. Additionally, 25.4% of the participants had hypertension, 17.1% had diabetes, and 30.6% experienced sleep disorders. A decreasing trend in the prevalence of depression was observed among the factors of interest in the study, with the prevalence significantly decreasing from 12.0% to 5.4% as serum ascorbic acid levels increased. Table 1 baseline characteristics for the total subjects displayed participants’ baseline characteristics.

**Table 1:** Baseline characteristics for the total subjects.

Characteristics	Total subjects	Serum ascorbic acid quartile, μmol/L			
		Quartile 1 (Q_1_) (<30.0)	Quartile 2 (Q_2_) (30.0–50.0)	Quartile 3 (Q_3_) (50.0–67.0)	Quartile 4 (Q_4_) (≥67.0)
***N***	3,404	853	851	855	845
**Gender, *n*(%)**					
Male	1739 (51.1)	499 (58.5)	482 (56.6)	453 (53.0)	305 (36.1)
Female	1,665 (48.9)	354 (41.5)	369 (43.4)	402 (47.0)	540 (63.9)
**Age, *n*(%)**					
20–39 years	1,026 (30.2)	240 (28.1)	283 (33.3)	276 (32.3)	227 (26.9)
40–59 years	1,093 (32.1)	294 (34.5)	289 (34.0)	290 (33.9)	220 (26.0)
≥60 years	1,285 (37.7)	319 (37.4)	279 (32.8)	289 (33.8)	398 (47.1)
**Race, *n*(%)**					
Mexican American	457 (13.4)	85 (10.0)	141 (16.6)	134 (15.7)	95 (11.5)
Other Hispanic	300 (8.8)	52 (6.1)	101 (11.9)	74 (8.7)	73 (8.6)
Non-Hispanic White	1,332 (39.1)	401 (47.0)	237 (27.8)	300 (35.1)	394 (46.6)
Non-Hispanic Black	750 (22.0)	212 (24.9)	209 (24.6)	183 (21.4)	146 (17.3)
Other Race	565 (16.6)	103 (12.0)	163 (19.2)	164 (19.2)	135 (16.0)
**Education levels, *n*(%)**					
Less than 12th grade	570 (16.7)	169 (19.8)	166 (19.5)	121 (14.2)	114 (13.5)
High school graduate	839 (24.6)	265 (31.0)	195 (22.9)	200 (23.4)	179 (21.2)
Some college or AA degree	1,163 (34.3)	300 (35.2)	289 (34.0)	284 (33.2)	290 (34.3)
College graduate or above	832 (24.4)	119 (14.0)	201 (23.6)	250 (29.2)	262 (31.0)
**Marital status, *n*(%)**					
Married/living with a partner	2022 (59.4)	479 (56.2)	522 (61.3)	534 (62.5)	487 (57.6)
Widowed/divorced/separated	800 (23.5)	220 (25.8)	178 (20.9)	178 (20.9)	224 (26.5)
Never married	582 (17.1)	154 (18.0)	151 (17.8)	143 (16.6)	134 (15.9)
**PIR, *n*(%)**					
Q_1_<1.24	854 (25.1)	271 (31.8)	223 (26.2)	186 (21.8)	174 (20.6)
Q_2_ 1.24–2.24	852 (25.0)	246 (28.8)	199 (23.4)	219 (25.6)	188 (22.2)
Q_3_ 2.25–3.98	852 (25.0)	175 (20.5)	228 (26.9)	213 (24.9)	236 (27.9)
Q_4_ ≥ 3.99	846 (24.9)	161 (18.9)	201 (23.6)	237 (27.7)	247 (29.3)
**BMI, kg/m** ^**2**^	29.60 ± 6.57	30.68 ± 6.98	30.31 ± 6.45	29.54 ± 6.48	27.85 ± 5.95
**WBC, 1000 cells/μL**	7.26 ± 2.15	7.61 ± 2.32	7.45 ± 2.12	7.01 ± 1.98	6.95 ± 2.09
**HSCRP, *n*(%)**					
<1.00	961 (28.2)	162 (19.0)	223 (26.2)	283 (33.1)	293 (34.7)
1.00–3.00	1,225 (36.0)	302 (35.4)	299 (35.1)	295 (34.5)	329 (38.9)
>3.00	1,218 (35.8)	389 (45.6)	329 (38.7)	277 (32.4)	223 (26.4)
**PHQ-9 score ≥ 10, *n*(%)**					
No	3,103 (91.2)	751 (88.0)	760 (89.3)	793 (92.7)	799 (94.6)
Yes	301 (8.8)	102 (12.0)	91 (10.7)	62 (7.3)	46 (5.4)
**Smoking status, *n*(%)**					
No	1820 (53.5)	317 (37.2)	476 (55.9)	496 (58.0)	531 (62.8)
Yes	1,584 (46.5)	536 (62.8)	375 (44.1)	359 (42.0)	364 (37.2)
**Alcohol status, *n*(%)**					
Never	779 (22.9)	235 (27.6)	188 (22.1)	161 (18.8)	195 (23.1)
No more than once a month	1,213 (35.6)	296 (34.7)	305 (35.8)	311 (36.4)	301 (35.6)
No more than once a week	700 (20.6)	134 (15.7)	177 (20.8)	192 (22.5)	197 (23.3)
More than once a week	712 (20.9)	188 (22.0)	181 (21.3)	191 (22.3)	152 (18.0)
**Hypertension, *n*(%)**					
No	2,540 (74.6)	626 (73.4)	618 (72.6)	663 (77.5)	633 (74.9)
Yes	864 (25.4)	227 (26.6)	233 (27.4)	192 (22.5)	212 (25.1)
**Diabetes, *n*(%)**					
No	2,823 (82.9)	683 (80.1)	698 (82.0)	722 (84.4)	720 (85.2)
Yes	587 (17.1)	170 (19.9)	153 (18.0)	133 (15.6)	125 (14.8)
**Trouble sleeping, *n*(%)**					
No	2,362 (69.4)	534 (62.6)	614 (72.2)	617 (72.2)	539 (70.7)
Yes	1,042 (30.6)	319 (37.4)	237 (27.8)	238 (27.8)	248 (29.3)

Percentages (%) for categorical variables, mean ± standard deviation (SD) for continuous variables. PIR, the ratio of family income to poverty; BMI, body mass index; WBC, white blood cell count; HSCRP, high-sensitivity C-reactive protein.

### 3.2 Analysis of depression risk factors pre and post matching

Based on the findings presented in Table 2, it was observed that among the initial sample of participants, a total of 301 individuals were diagnosed with depression. The results of the chi-square test revealed a significant association between depression and various confounding factors, excluding age, race, and hypertension (*p* < 0.05). To further affirm the association between serum ascorbic acid levels and the prevalence of depression, a well-matched control group was established using PSM with a 1:4 ratio and a caliper value of 0.02. This matching approach allowed us to control for potential confounding factors, including demographic variables such as gender, age, race, education levels, marital status, and PIR.

**Table 2:** Variables of full and propensity score matched cohorts by PHQ-9.

Variables	Before matching			After matching		
	Control group	Depression group	*p* value	Control group	Depression group	*p* value
***N***	3,103 (91.2)	301 (8.8)		1,107 (78.7)	299 (21.3)	
**Gender, *n*(%)**			**<0.001**			0.487
Men	1,619 (52.2)	120 (39.9)		469 (42.4)	120 (40.1)	
Women	1,484 (47.8)	181 (60.1)		638 (57.6)	179 (59.9)	
**Age, *n*(%)**			0.264			0.285
20–39 years	932 (30.0)	94 (31.2)		354 (32.0)	93 (31.1)	
40–59 years	987 (31.8)	106 (35.2)		338 (30.5)	105 (35.1)	
≥60 years	1,184 (38.2)	101 (33.6)		415 (37.5)	101 (33.8)	
**Race, *n*(%)**			0.446			0.323
Mexican American	420 (13.5)	37 (12.3)		137 (12.4)	37 (12.4)	
Other Hispanic	274 (8.9)	26 (8.6)		86 (7.8)	25 (8.4)	
Non-Hispanic White	1,199 (38.6)	133 (44.2)		463 (41.8)	132 (44.1)	
Non-Hispanic Black	691 (22.3)	59 (19.6)		280 (25.3)	59 (19.7)	
Other races	519 (16.7)	46 (15.3)		141 (12.7)	46 (15.4)	
**Education levels, *n*(%)**			**<0.001**			0.586
Less than 12th grade	502 (16.2)	68 (22.6)		219 (19.8)	66 (22.1)	
High school graduate	750 (24.2)	89 (29.6)		363 (32.8)	89 (29.8)	
Some college or AA degree	1,058 (34.0)	105 (34.8)		365 (33.0)	105 (35.1)	
College graduate or above	793 (25.6)	39 (13.0)		160 (14.4)	39 (13.0)	
**Marital status, *n*(%)**			**<0.001**			0.203
Married/living with a partner	1890 (60.9)	132 (43.9)		541 (48.9)	132 (44.1)	
Widowed/divorced/separated	694 (22.4)	106 (35.2)		331 (29.9)	105 (35.1)	
Never married	519 (16.7)	63 (20.9)		235 (21.2)	62 (20.8)	
**PIR, *n*(%)**			**<0.001**			0.441
Q_1_<1.23	738 (23.8)	116 (38.5)		384 (34.7)	114 (38.1)	
Q_2_ 1.23–2.24	764 (24.6)	88 (29.2)		332 (30.0)	88 (29.4)	
Q_3_ 2.25–4.25	796 (25.7)	56 (18.6)		251 (22.7)	56 (18.7)	
Q_4_ ≥ 4.26	805 (25.9)	41 (13.6)		140 (12.6)	41 (13.7)	
**Serum ascorbic acid, mol/L**	50.97 ± 27.25	42.97 ± 30.64	**<0.001**	49.28 ± 28.30	43.08 ± 30.67	**<0.001**
**Serum ascorbic acid, *n*(%)**			**<0.001**			**<0.001**
Q_1_<30.0	751 (24.2)	102 (33.9)		311 (28.1)	101 (33.8)	
Q_2_ 30.0–49.9	760 (24.5)	91 (30.2)		248 (22.4)	90 (30.1)	
Q_3_ 50.0–66.9	793 (25.6)	62 (20.6)		268 (24.2)	62 (20.7)	
Q_4_ ≥ 67.0	799 (25.7)	46 (15.3)		280 (25.3)	46 (15.4)	
**BMI, kg/m** ^**2**^	29.49 ± 6.50	30.76 ± 7.12	**0.001**	29.68 ± 6.95	30.73 ± 7.11	**0.022**
**WBC, 1000 cells/μL**	7.21 ± 2.11	7.76 ± 2.48	**<0.001**	7.36 ± 2.14	7.76 ± 2.48	**0.006**
**HSCRP, *n*(%)**			**<0.001**			0.070
<1.00	896 (28.9)	65 (21.6)		297 (26.8)	65 (21.7)	
1.00–3.00	1,126 (36.3)	99 (32.9)		382 (34.5)	98 (32.8)	
>3.00	1,081 (34.8)	137 (45.5)		428 (38.7)	136 (45.5)	
**Smoking status, *n*(%)**			**<0.001**			**<0.001**
No	1,699 (54.8)	121 (40.2)		579 (52.3)	121 (40.5)	
Yes	1,404 (45.2)	180 (59.8)		528 (47.7)	158 (59.5)	
**Alcohol status, *n*(%)**			**0.011**			0.322
Never	690 (22.2)	89 (29.6)		279 (25.2)	89 (29.8)	
No more than once a month	1,105 (35.6)	108 (35.8)		414 (37.4)	108 (36.1)	
No more than once a week	654 (21.1)	46 (15.3)		208 (18.8)	46 (15.4)	
More than once a week	654 (21.1)	58 (19.3)		206 (18.6)	56 (18.7)	
**Hypertension, *n*(%)**			0.637			0.254
No	2,312 (74.5)	228 (75.7)		804 (72.6)	227 (75.9)	
Yes	791 (25.5)	73 (24.3)		302 (27.4)	72 (24.1)	
**Diabetes, *n*(%)**			**0.012**			**0.035**
No	2,589 (83.4)	234 (77.7)		921 (83.2)	233 (77.9)	
Yes	514 (16.6)	67 (22.3)		186 (16.8)	66 (22.1)	
**Trouble sleeping, *n*(%)**			**<0.001**			**<0.001**
No	2,258 (72.8)	104 (34.6)		779 (70.4)	102 (34.1)	
Yes	845 (27.2)	197 (65.4)		328 (29.6)	197 (65.9)	

Percentages (%) for categorical variables, mean ± standard deviation (SD) for continuous variables. PIR, the ratio of family income to poverty; BMI, body mass index; WBC, white blood cell count; HSCRP, high-sensitivity C-reactive protein.

Ultimately, 1,107 participants were successfully included in the control group, and 299 participants in the depression group, ensuring a balanced comparison between the two groups. Consistent with our expectations, the post-matching results revealed non-significant effects of the demographic confounding factors (*p* > 0.05), while significant associations persisted for other confounding factors such as serum ascorbic acid, WBC, BMI, smoking status, diabetes, and trouble sleeping. Notably, the results after matching showed that the prevalence of depression decreased from 33.8 to 15.4% in the case group with the increase of serum ascorbic acid level, while the prevalence of the control group was maintained at 25% ± 3%. The results of serum ascorbic acid levels, whether analyzed as continuous variables or categorical variables, exhibited statistically significant significance (*p* < 0.001).

### 3.3 Higher serum ascorbic acid levels linked to lower depression prevalence

**Figure 2:**
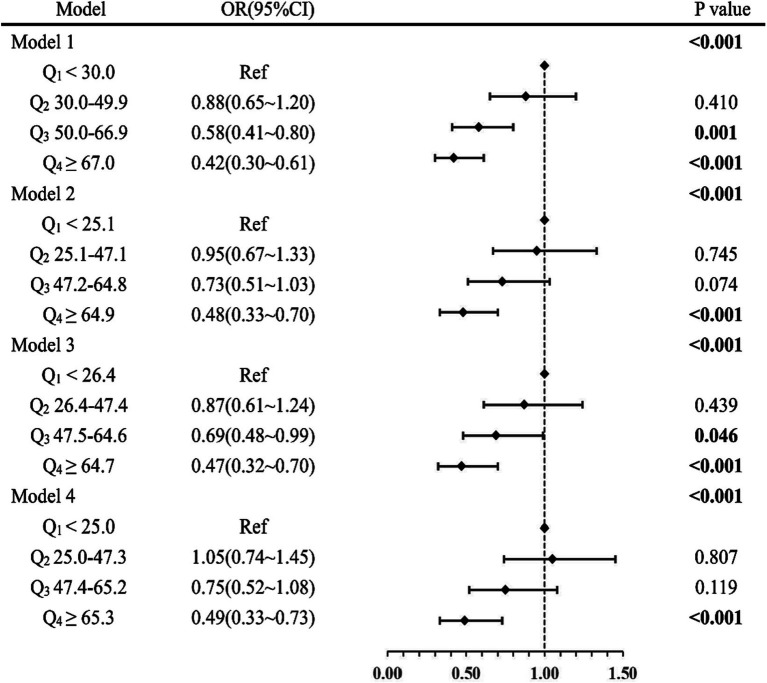
Hierarchical comparison of models forest plot. OR, odds ratio; 95%CI, 95% confidence interval. Model 1: adjusted for no covariates. Model 2: adjusted for gender, age, race, education levels, marital status, and PIR. Model 3: adjusted for Model 2 covariates and for smoking status, alcohol status, hypertension, diabetes, and trouble sleeping. Model 4: adjusted for all covariates.

Multiple models for binary logistic regression analysis were established to investigate the relationship between serum ascorbic acid levels and the prevalence of depression. The results, as depicted in Figure 2.

Hierarchical comparison of models forest plot. Indicated statistical significance (*p* < 0.001) for all four models considered in the analysis.

In Model 1, participants with serum ascorbic acid levels in Q_4_ were observed to have a lower risk of depression compared to those in Q_1_, with an odds ratio of 0.42 (95%CI: 0.30 ~ 0.61, *p* < 0.001). Similar results were obtained in Model 2 (Q_4_ vs. Q_1_: OR = 0.48; 95%CI: 0.33 ~ 0.70; *p* < 0.001) and Model 3 (Q_4_ vs. Q_1_: OR = 0.47; 95%CI: 0.32 ~ 0.70; *p* < 0.001). In the final Model 4, after adjusting for all confounding factors, higher serum ascorbic acid levels remained significantly associated with a reduced prevalence of depression (Q_4_ vs. Q_1_: OR = 0.49; 95%CI: 0.33 ~ 0.73; *p* < 0.001). These consistent findings across the four models indicated a significant negative association between higher serum ascorbic acid levels and a lower depression prevalence.

### 3.4 Restricted cubic splines before and after matching

To visualize the relationship between serum ascorbic acid levels and the prevalence of depression, RCS functions were employed for a more intuitive presentation of the dose–response connection. This method allowed for the visual exploration of the potential non-linear association between serum ascorbic acid levels and the prevalence of depression. Simultaneously, the introduction of linear splines was employed to mitigate the issue of overfitting caused by RCS, thereby simulating the linear relationship between the two variables. This integrated utilization of diverse fitting methods facilitates a comprehensive and precise comprehension of the intricate association between serum ascorbic acid and depression risk.

**Figure 3:**
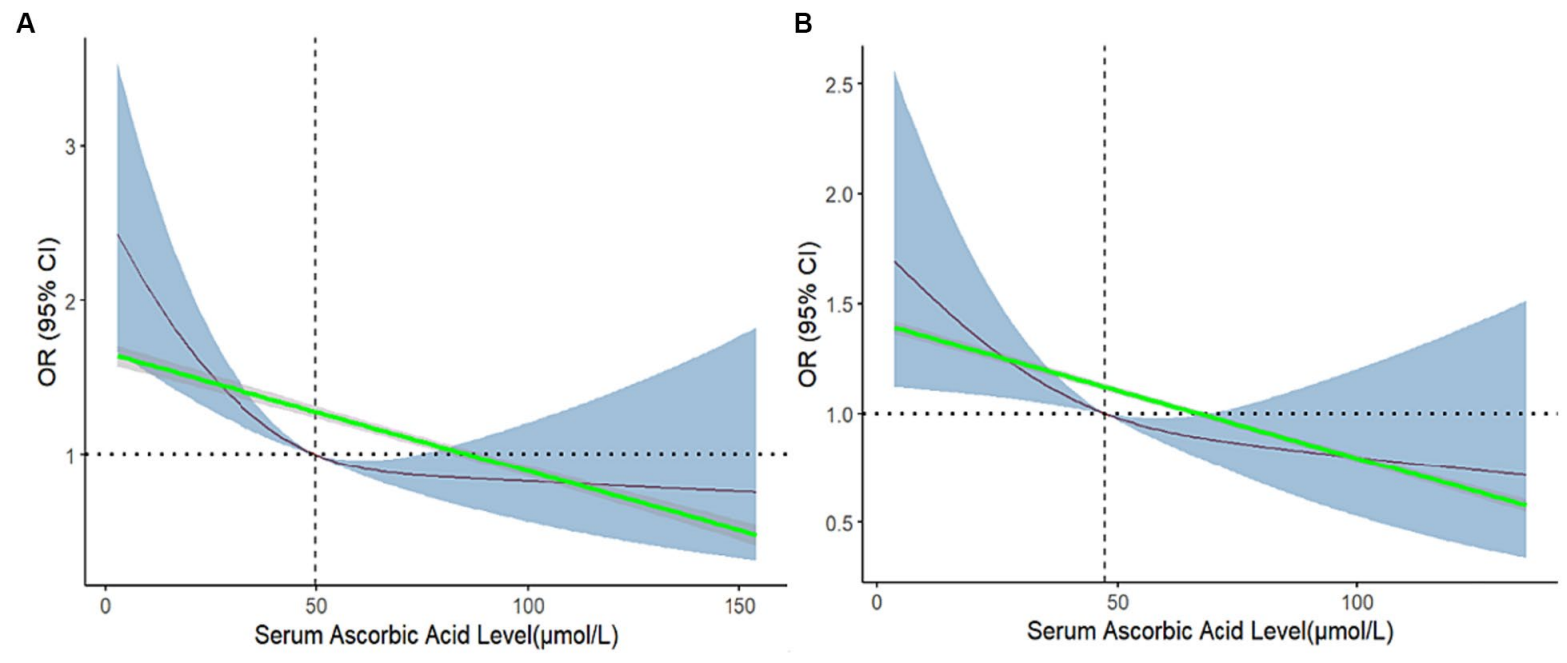
Restricted cubic spline and linear spline for odds ratios of depression with serum ascorbic acid before and after matching. The 95%CI of the OR in the restricted cubic spline is visually represented by the blue-shaded area, while the gray-shaded area represents the 95%CI of the OR in the linear splines. In **(A)**, no confounding factors were adjusted, and in **(B)**, all confounding factors were adjusted.

In Figure 3A without adjustments, a notably significant L-shaped non-linear relationship was observed between serum ascorbic acid levels concentration and the depression prevalence (P for nonlinearity <0.05). As serum ascorbic acid levels concentration increased, the prevalence of depression showed a clear decreasing trend, suggesting a potential dose-dependent protective effect. An interesting finding was the intersection of the curve with the reference line (OR = 1) occurring at a serum ascorbic acid level of 49.9, which perfectly coincided with the median of serum ascorbic acid levels before adjustment. The linear splines demonstrated a consistent and significant downward trend, indicating that there was a negative association between serum ascorbic acid level and the risk of depression. Emphasizing a potential threshold effect, this observation indicated that maintaining higher serum ascorbic acid levels could be linked to a lowered prevalence of depression.

After matching for all confounding factors, the previously observed non-linear relationship (P for nonlinear >0.05) had disappeared, and the curve had become smoother. Importantly, the overall trend of the restricted cubic spline and linear spline remained consistent with the pre-matched analysis, indicating a negative correlation between serum ascorbic acid levels concentration and the prevalence of depression. Figure 3 provided a detailed comparison.

## 4 Discussion

Our study investigated the relationship between serum ascorbic acid levels and the prevalence of depression using data from the 2017–2018 NHANES. The results revealed that the serum ascorbic acid levels of depressed patients were significantly lower than those of non-depressed individuals (42.97 *VS* 52.97 μmol/L). Even after employing PSM to control for potential confounding variables, the chi-square tests still revealed a significant correlation between serum ascorbic acid levels and depression prevalence. Similarly, the forest plot results from four models and the RCS plots before and after matching further substantiated this conclusion. Additionally, regardless of whether serum ascorbic acid was treated as a continuous numerical value or categorized into different groups, the results demonstrated statistical significance. These findings provided evidence to suggest that maintaining high serum ascorbic acid levels may potentially help reduce the prevalence of depression.

It is noteworthy that our research findings have also received support from other studies. After comprehensive adjustment for multiple variables, a meta-analysis summarizing 25 studies involving 91,966 participants revealed a negative correlation between dietary vitamin C intake and depressive symptoms (RR = 0.72, 95% CI: 0.57 ~ 0.91; *p* = 0.005) (28). This conclusion was further supported by another cross-sectional study. The results indicated that among middle-aged American women, the adjusted OR and 95% CI for depressive symptoms were 0.699 (0.52 ~ 0.93) in Q_4_ compared to Q_1_ of ascorbic acid intake (9). Furthermore, the findings of Wang et al. also demonstrate a negative correlation between dietary intake of vitamin C and vegetable-derived vitamin C with the risk of depressive symptoms in the general population, suggesting an increase in vegetable consumption as part of a balanced diet (29).

Numerous studies were currently investigating the potential role and mechanisms of ascorbic acid in depression. Oxidative stress significantly contributed to the onset of depression (30, 31), and as demonstrated, the escalation of oxidative stress can serve as a triggering factor for the decline in neurotransmitter function, thereby precipitating the manifestation of depressive behavior (32). As a crucial antioxidant, AA deficiency often results in the impairment of antioxidant function within the body, consequently leading to an elevation in oxidative stress levels. Concerning this, glucocorticoids, as stress hormones, enhance the expression of key subunits p47phox and p67phox of NADPH oxidase in the brain by upregulating superoxide production via NADPH oxidase (33). This mechanism ultimately mitigates stress-induced depression. The findings of population-based studies have also demonstrated a significant elevation in baseline levels of oxidative stress markers among individuals with depression compared to those without, indicating a strong association between depression and heightened oxidative stress as evidenced by elevated serum prooxidant-antioxidant balance (34, 35).

The dysfunction of neurotrophins, particularly the decreased functioning of brain-derived neurotrophic factors, is believed to be a consequence of oxidative stress triggering (36, 37). Elevated levels of oxidative stress disrupt the normal functioning of neurotransmitters and directly impact mood and depressive states. Oxidative stress promotes the progression of depression by inducing damage and degenerative changes in brain tissue, resulting in the inactivation of neurotransmitters such as acetylcholine and monoamine neurotransmitters under conditions of oxidative stress (38, 39). Therefore, modulation of the monoaminergic and glutamatergic neurotransmitter systems is frequently regarded as a crucial target for achieving the antidepressant and anxiolytic effects of AA (40). The neuroprotective effect of AA is complemented by its involvement in neurotransmitter synthesis and the secretion of lipid mediators, playing a pivotal role in the epigenetic regulation of DNA (39, 41). When cyclooxygenase (COX)-2 is highly expressed in the dentate gyrus (DG) of the hippocampus, the resulting oxidative stress is significantly associated with depression-like behaviors. Moreover, inhibition of COX-2 activity in the DG area by oxidative stress weakening inhibitors leads to a noticeable improvement in depressive behavior. This can be attributed to the neuroprotective effects of inhibiting COX-2 activity on the DG region, which includes suppressing neuroinflammatory responses, mitigating oxidative stress, and preventing neuronal apoptosis—all crucial risk factors for neuronal damage and depression pathology.

It is crucial to acknowledge certain limitations in our study. On one hand, the inclusion of dietary ascorbic acid intakes was not considered in our study to examine the potential non-correlation between dietary intakes and serum levels, thereby supporting the notion that there may not always be a direct relationship between dietary intakes and serum ascorbic acid contents. On the other hand, due to the relatively low prevalence of depression in our study and the limitations of NHANES data, more detailed classifications of depression could not be performed, and only a simple binary analysis was conducted. In addition, the data results of our study only pertain to the general adult population in the United States, thus limiting its applicability to a global context. Future research could consider incorporating dietary factors to comprehensively assess their influence on the prevalence of depression, as well as investigate in depth the associations between varying degrees of depressive symptoms and serum ascorbic acid levels.

## 5 Conclusion

The results demonstrated a negative correlation between serum ascorbic acid levels and depression, indicating a significant association between higher serum ascorbic acid levels and a lower prevalence of depression. This study provided valuable insights regarding the potential application of serum ascorbic acid in the prevention and treatment of depression, and the findings shed light on significant implications for advancing mental health research and refining strategies for depression prevention and management.

## Data availability statement

The datasets presented in this study can be found in online repositories. The names of the repository/repositories and accession number(s) can be found in the article/supplementary material.

## Ethics statement

The studies involving humans were approved by the Institutional Review Board of the Centers for Disease Control. The studies were conducted in accordance with the local legislation and institutional requirements. The participants provided their written informed consent to participate in this study. Written informed consent was obtained from the individual(s) for the publication of any potentially identifiable images or data included in this article.

## Author contributions

MC: Conceptualization, Data curation, Formal analysis, Investigation, Methodology, Project administration, Software, Validation, Visualization, Writing – original draft. HL: Conceptualization, Data curation, Formal analysis, Writing – original draft. YH: Funding acquisition, Supervision, Writing – review & editing. YL: Software, Supervision, Validation, Visualization, Writing – original draft. LZ: Investigation, Methodology, Project administration, Writing – original draft. XR: Funding acquisition, Resources, Supervision, Writing – review & editing.
